# Supplementary material for: Tick-Borne Pathogens Shape the Native Microbiome Within Tick Vectors
Source: Microorganisms. 2020 Aug 25;8(9):1299. doi: 10.3390/microorganisms8091299 (PMC7563471; doi:10.3390/microorganisms8091299)
Supplement: Supplementary file 1 [file microorganisms-08-01299-s001.pdf]

SUPPLEMENTARY DATA

Table S1: Total number of ticks used in this study and the percentage of those identified with *A. marginale, Theileria*, and *Wolbachia* based on PCR analysis and amplicon sequencing.

Number of ticks PCR positive for *Theileria*, *A. marginale*, and *Wolbachia* in the ticks studied				
Tick Specie	Size	*A. marginale* positive	*Theileria* positive	*Wolbachia* positive
H. anatolicum	198	2.6%(n=5/198)	36.4%(n=72/198)	0.5%(n=1/198)
R. microplus	122	16.4%(n=20/122)	14.8%(n=18/122)	1.6%(n=2/122)
Total	320	7.2%(n=23/320)	28.1%(n=90/320)	0.9%(n=3/320)

Table S2: Total number of reads that used in the analysis after filtering and removal of chimeras in *H. anatolicum* ticks.

	Input	Filtered	DenoisedF	DenoisedR	Merged	Nonchim
**HA-S1**	115207	62238	61212	60891	6697	6643
**HA-S10**	42648	22866	21589	21109	7559	7447
**HA-S11**	90600	48598	47911	47198	1624	1623
**HA-S12**	123736	63979	62900	62015	7151	6947
**HA-S13**	48407	25292	24096	23842	13079	12828
**HA-S14**	64705	32573	31796	30919	3970	3964
**HA-S15**	59703	31158	30682	30172	819	817
**HA-S16**	113459	63509	62572	62318	13999	12997
**HA-S17**	45592	23449	22469	22141	9763	9641
**HA-S18**	17067	8443	7850	7801	2970	2959
**HA-S19**	49866	27339	26938	26218	3567	3548
**HA-S2**	86522	47517	46549	46267	9723	9628
**HA-S20**	55424	32722	31936	31582	10188	10139
**HA-S3**	56262	30303	29485	29210	5067	5005
**HA-S4**	39280	17445	15961	15571	7706	7663
**HA-S5**	64392	32428	31626	31253	3708	3657
**HA-S6**	61695	31410	31197	30659	29	29
**HA-S7**	51702	28486	28012	27650	2514	2513
**HA-S8**	86403	51321	50708	50293	2407	2394
**HA-S9**	36124	19123	18622	18317	3592	3585

Table S3: Total number of reads that used in the analysis after filtering and removal of chimeras in *R. microplus* ticks

	**input**	**filtered**	**denoisedF**	**denoisedR**	**merged**	**nonchim**
**RM-S21**	95301	35530	35290	35349	499	495
**RM-S22**	108550	38281	38053	38063	328	328
**RM-S23**	96202	35826	35628	35617	387	373
**RM-S24**	66513	24083	23901	23832	213	213
**RM-S25**	56080	17690	17521	17507	123	109
**RM-S26**	54840	18752	18363	18113	260	260
**RM-S27**	26575	9262	8980	8804	214	213
**RM-S28**	58178	20095	19878	19542	138	138
**RM-S29**	74435	28375	27836	27586	224	220
**RM-S30**	97340	35873	35371	35104	1001	994
**RM-S31**	78132	28127	27683	27395	363	363
**RM-S32**	115551	41676	41004	40952	219	210
**RM-S33**	107035	37570	37033	36773	519	512
**RM-S34**	78966	29770	29464	29122	955	948
**RM-S35**	45377	17773	17397	17248	694	694
**RM-S36**	107671	40301	39558	39473	4041	3829
**RM-S37**	47369	17903	17650	17431	219	218
**RM-S38**	110700	38914	38245	38153	1245	1238
**RM-S39**	79874	29785	29338	28973	572	565
**RM-S40**	89539	33312	32905	32665	508	498

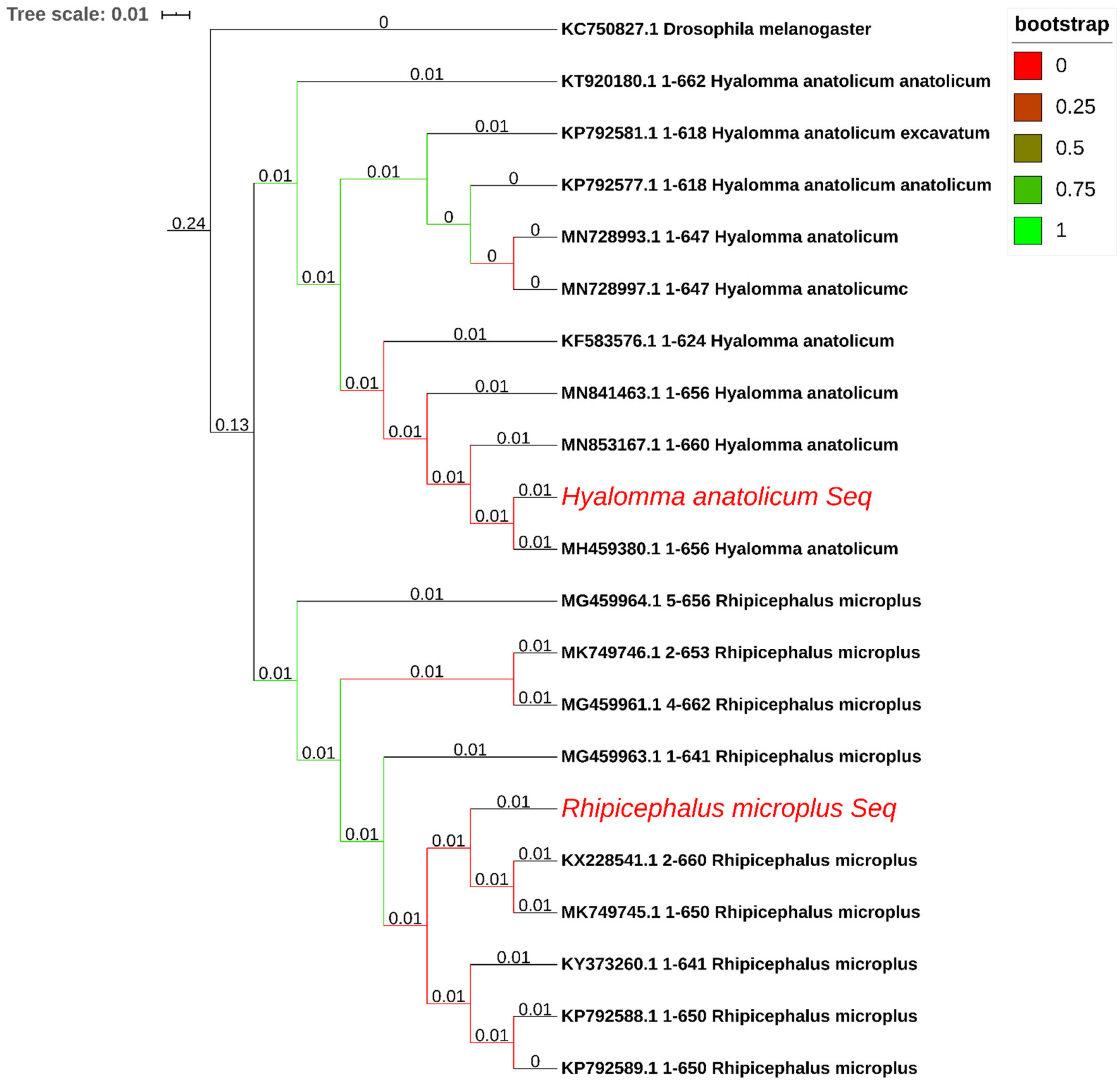

Figure S1: Phylogenetic analysis of amplified COI sequences of R. microplus and H. anatolicum ticks with other arthropod's COI genes from NCBI. Phylogenetic trees was evaluated using the UPGMA method and a bootstrap consensus of 1000 replicates. The *Drosophila melanogaster* cytochrome oxidase I (COI) gene was used as an out-group

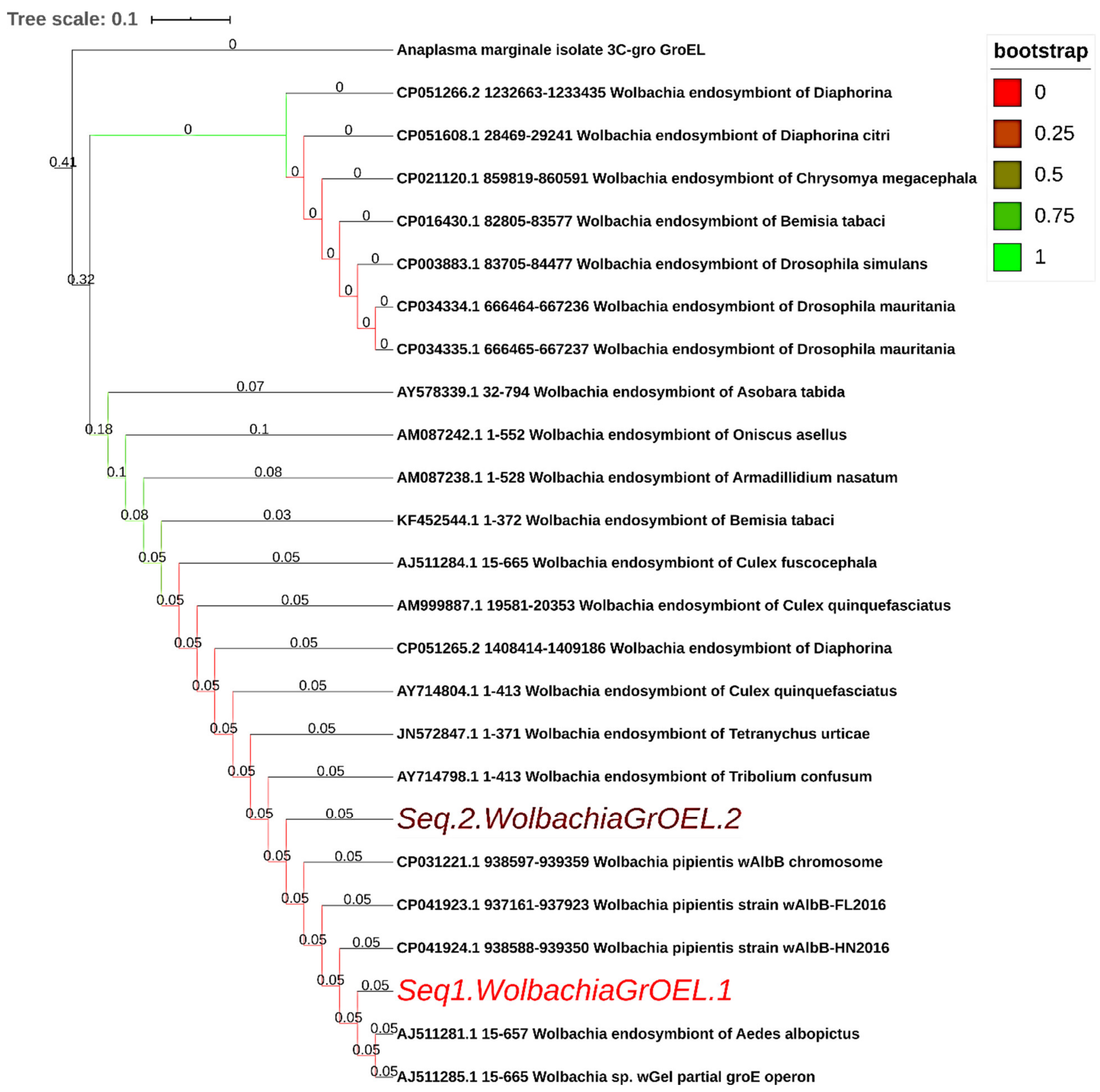

Figure S2: Genetic relationship of identified Wolbachia GroEL genes compared with selected sequences from NCBI. The 800bp sequences of the GroEL genes was analyzed using Neighbor Joining method. The *Anaplasma marginale* GroEL gene was used as the out-group.

Description	Max Score	Total Score	Query Cover	E value	Per. Ident	Accession
Wolbachia pipientis strain wAlbB-HN2016 chromosome, complete genome	1393	1393	99%	0.0	99.61%	CP041924.1
Wolbachia pipientis strain wAlbB-FL2016 chromosome, complete genome	1393	1393	99%	0.0	99.61%	CP041923.1
Wolbachia pipientis wAlbB chromosome, complete genome	1387	1387	99%	0.0	99.48%	CP031221.1
Wolbachia endosymbiont of Chrysomya megacephala isolate wMeg chromosome, complete genome	1303	1303	99%	0.0	97.29%	CP021120.1
Wolbachia endosymbiont of Diaphorina citri isolate dawsonii chromosome, complete genome	1303	1303	99%	0.0	97.29%	CP051608.1
Wolbachia endosymbiont of Diaphorina citri isolate KPSwDI05P26 chromosome	1303	1303	99%	0.0	97.29%	CP051266.2
Wolbachia endosymbiont of Diaphorina citri isolate KPSwDI10P38 chromosome	1303	1303	99%	0.0	97.29%	CP051265.2
Wolbachia endosymbiont of Diaphorina citri isolate KPSwDI15P40 chromosome	1303	1303	99%	0.0	97.29%	CP051264.2
Wolbachia endosymbiont of Culex quinquefasciatus Pel strain wPip complete genome	1303	1303	99%	0.0	97.29%	AM999887.1
Wolbachia endosymbiont of Drosophila mauritiana strain wMau chromosome, complete genome	1297	1297	99%	0.0	97.16%	CP034335.1
Wolbachia endosymbiont of Drosophila mauritiana strain wMau chromosome, complete genome	1297	1297	99%	0.0	97.16%	CP034334.1
Wolbachia endosymbiont of Drosophila simulans wNo, complete genome	1297	1297	99%	0.0	97.16%	CP003883.1
Wolbachia endosymbiont of Bemisia tabaci strain China 1 genome	1291	1291	99%	0.0	97.03%	CP016430.1
Wolbachia sp. group B genes for GroES protein homolog, GroEL protein homolog, partial cds	1273	1273	96%	0.0	97.47%	AB002290.1
Wolbachia sp. group B gene for GroES protein homolog, GroEL protein homolog, complete cds	1269	1269	99%	0.0	96.51%	AB002286.1
Wolbachia sp. wDry gene for molecular chaperone groES and groEL, partial cds	1267	1267	96%	0.0	97.34%	AB039282.1
Wolbachia sp. wJapo genes for molecular chaperone gro EL and groES, partial cds	1262	1262	96%	0.0	97.21%	AB039281.1

Figure S3: Blast results of the percentage identity and query cover of *Wolbachia* GroEL sequence from the current study when compared to previously deposited GroEL sequences from NCBI

Description	Max Score	Total Score	Query Cover	E value	Per. Ident	Accession
Hyalomma excavatum cytochrome oxidase subunit I (cox1) gene, partial cds; mitochondrial	1223	1223	96%	0.0	100.00%	KX911989.1
Hyalomma anatolicum isolate GY44-2 cytochrome oxidase subunit I (COI) gene, partial cds; mitochondrial	1203	1203	96%	0.0	99.55%	MN853167.1
Hyalomma detritum detritum isolate D1 cytochrome oxidase subunit I (Cox1) gene, partial cds; mitochondrial	1203	1203	95%	0.0	100.00%	KP792595.1
Hyalomma detritum detritum isolate D3 cytochrome oxidase subunit I (Cox1) gene, partial cds; mitochondrial	1203	1203	95%	0.0	100.00%	KP792574.1
Hyalomma anatolicum anatolicum isolate Gansu cytochrome oxidase subunit I (COI) gene, partial cds; mitochondrial	1203	1203	96%	0.0	99.55%	JQ737067.1
Hyalomma detritum detritum isolate Z6 cytochrome oxidase subunit I (Cox1) gene, partial cds; mitochondrial	1197	1197	94%	0.0	100.00%	KP792592.1
Hyalomma anatolicum anatolicum cytochrome oxidase subunit I gene, partial cds; mitochondrial	1195	1195	96%	0.0	99.24%	KT920180.1
Hyalomma anatolicum clone HS/C/170 cytochrome c oxidase subunit 1 gene, partial cds; mitochondrial	1195	1195	94%	0.0	100.00%	MN728993.1
Hyalomma detritum detritum isolate D2 cytochrome oxidase subunit I (Cox1) gene, partial cds; mitochondrial	1192	1192	94%	0.0	99.85%	KP792596.1
Hyalomma anatolicum voucher AC9 cytochrome oxidase subunit 1 (COI) gene, partial cds; mitochondrial	1190	1190	96%	0.0	99.39%	MH459380.1
Hyalomma anatolicum clone JH/B/139E cytochrome c oxidase subunit 1 gene, partial cds; mitochondrial	1190	1190	94%	0.0	99.85%	MN728996.1
Hyalomma anatolicum clone OK/C/42 cytochrome c oxidase subunit 1 gene, partial cds; mitochondrial	1190	1190	94%	0.0	99.85%	MN728995.1
Hyalomma anatolicum clone SU/B/332 cytochrome c oxidase subunit 1 gene, partial cds; mitochondrial	1190	1190	94%	0.0	99.85%	MN728994.1
Hyalomma anatolicum clone HS/C/172E cytochrome c oxidase subunit 1 gene, partial cds; mitochondrial	1190	1190	94%	0.0	99.85%	MN728992.1
Hyalomma anatolicum clone OK/C/91 cytochrome c oxidase subunit 1 gene, partial cds; mitochondrial	1190	1190	94%	0.0	99.85%	MN728991.1
Hyalomma anatolicum isolate GY43-1 cytochrome oxidase subunit I (COX1) gene, partial cds; mitochondrial	1184	1184	96%	0.0	99.24%	MN841463.1
Hyalomma anatolicum voucher AC5 cytochrome oxidase subunit 1 (COI) gene, partial cds; mitochondrial	1184	1184	96%	0.0	99.24%	MH459377.1
Hyalomma anatolicum clone SU/C/399 cytochrome c oxidase subunit 1 gene, partial cds; mitochondrial	1184	1184	94%	0.0	99.69%	MN728997.1
Hyalomma anatolicum voucher ACc cytochrome oxidase subunit 1 (COI) gene, partial cds; mitochondrial	1179	1179	96%	0.0	99.09%	MH459383.1
Hyalomma anatolicum voucher ACa cytochrome oxidase subunit 1 (COI) gene, partial cds; mitochondrial	1179	1179	96%	0.0	99.09%	MH459382.1
Hyalomma anatolicum voucher AC2 cytochrome oxidase subunit 1 (COI) gene, partial cds; mitochondrial	1179	1179	96%	0.0	99.09%	MH459375.1
Hyalomma anatolicum voucher AC3 cytochrome oxidase subunit 1 (COI) gene, partial cds; mitochondrial	1173	1173	96%	0.0	98.93%	MH459376.1
Hyalomma anatolicum isolate XJ187 cytochrome c oxidase subunit I gene, partial cds; mitochondrial	1153	1153	92%	0.0	99.53%	KC203438.1

Description	Max Score	Total Score	Query Cover	E value	Per. Ident	Accession
Rhipicephalus microplus isolate NIVEDI_2019_PK04 cytochrome oxidase subunit 1 (COI) gene, partial cds; mitochondrial	1173	1173	95%	0.0	98.48%	MK749744.1
Rhipicephalus microplus cytochrome c oxidase subunit 1 (cox1) gene, partial cds; mitochondrial	1173	1173	95%	0.0	98.48%	KX228541.1
Rhipicephalus microplus isolate 3 cytochrome oxidase subunit I (Cox1) gene, partial cds; mitochondrial	1171	1171	94%	0.0	98.92%	KP792588.1
Rhipicephalus microplus isolate BD1 cytochrome oxidase subunit 1 (COI) gene, partial cds; mitochondrial	1168	1168	95%	0.0	98.33%	MG459961.1
Rhipicephalus microplus isolate NIVEDI_2019_PK05 cytochrome oxidase subunit 1 (COI) gene, partial cds; mitochondrial	1166	1166	94%	0.0	98.77%	MK749745.1
Rhipicephalus microplus isolate NIVEDI_2019_PK07 cytochrome oxidase subunit 1 (COI) gene, partial cds; mitochondrial	1164	1164	94%	0.0	98.62%	MK749746.1
Rhipicephalus microplus isolate 4 cytochrome oxidase subunit I (Cox1) gene, partial cds; mitochondrial	1160	1160	94%	0.0	98.62%	KP792589.1
Rhipicephalus microplus isolate MN1 cytochrome oxidase subunit 1 (COI) gene, partial cds; mitochondrial	1158	1158	94%	0.0	98.47%	MG459964.1
Rhipicephalus microplus isolate PK1 cytochrome oxidase subunit 1 (COI) gene, partial cds; mitochondrial	1158	1158	92%	0.0	99.06%	MG459963.1
Rhipicephalus microplus cytochrome oxidase subunit 1 gene, partial cds; mitochondrial	1158	1158	92%	0.0	99.06%	KY373260.1
Rhipicephalus microplus isolate BD2 cytochrome oxidase subunit 1 (COI) gene, partial cds; mitochondrial	1157	1157	95%	0.0	98.03%	MG459962.1
Rhipicephalus microplus isolate 5 cytochrome oxidase subunit I (Cox1) gene, partial cds; mitochondrial	1157	1157	92%	0.0	99.22%	KP792580.1
Rhipicephalus microplus isolate NIVEDI_2019_PK01 cytochrome oxidase subunit 1 (COI) gene, partial cds; mitochondrial	1155	1155	92%	0.0	99.37%	MK736267.1
Rhipicephalus microplus cytochrome oxidase subunit I (Cox1) gene, partial cds; mitochondrial	1151	1151	92%	0.0	99.06%	KP792579.1
Rhipicephalus microplus isolate Z11 cytochrome oxidase subunit I (Cox1) gene, partial cds; mitochondrial	1149	1149	92%	0.0	99.21%	KP792578.1
Rhipicephalus microplus voucher RO_Rhphimic1 cytochrome c oxidase subunit 1 (Cox1) gene, partial cds; mitochondrial	1149	1149	92%	0.0	99.06%	KP318133.1
Rhipicephalus microplus isolate 1 cytochrome oxidase subunit I (Cox1) gene, partial cds; mitochondrial	1147	1147	91%	0.0	99.21%	KP792586.1

Figure S4: Blast results of the percentage identity and query cover of *Hyalomma anatolicum* and *Rhipicephalu microplus* COI sequence from the current study when compared to previously deposited COI sequences from NCBI

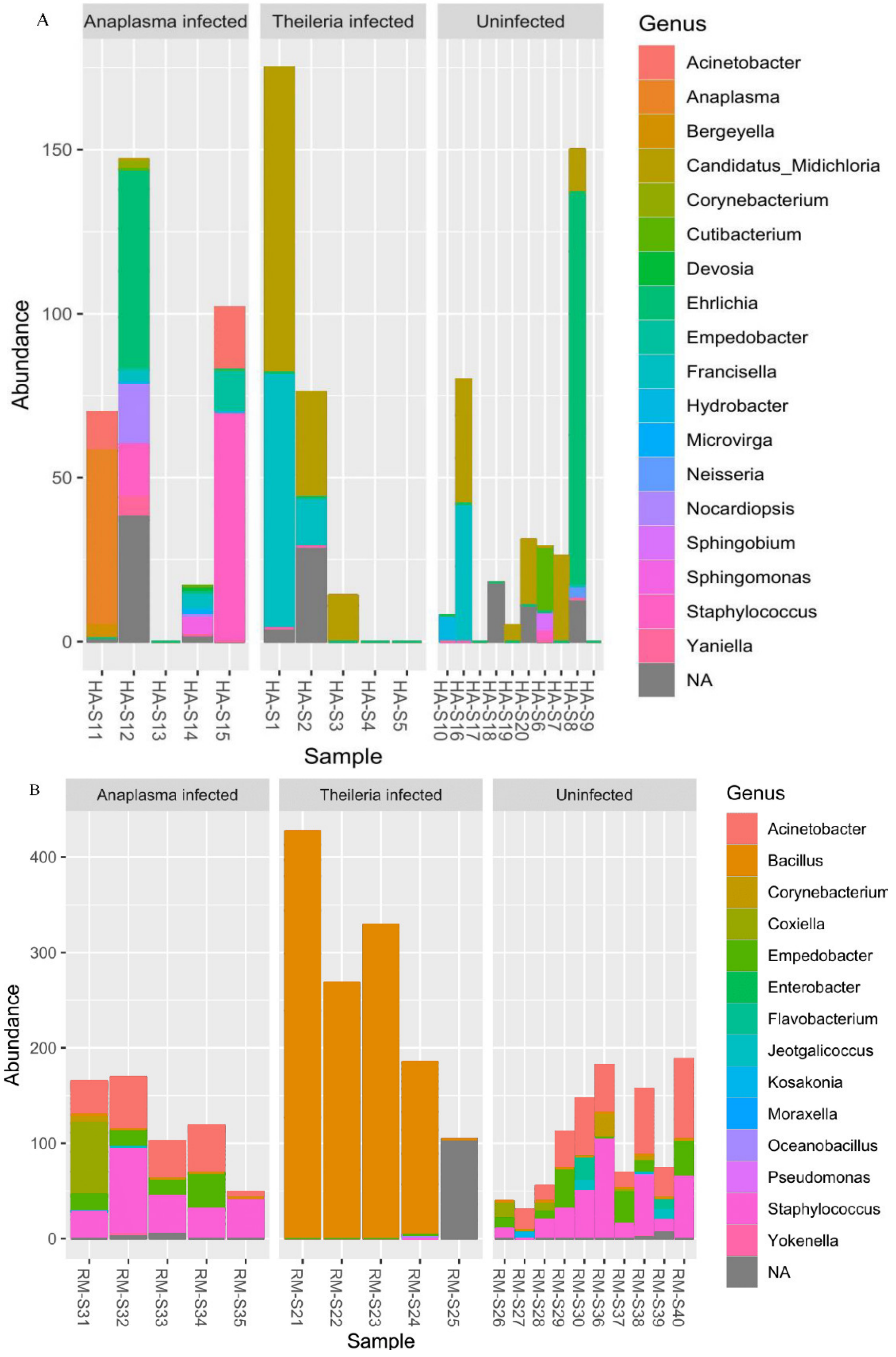

Figure S5: Relative abundances of bacteria species from individual tick samples used in this study. (A) Bacteria species abundance in *H. anatolicum* ticks. (B) Bacteria species abundance in *R. microplus* ticks.

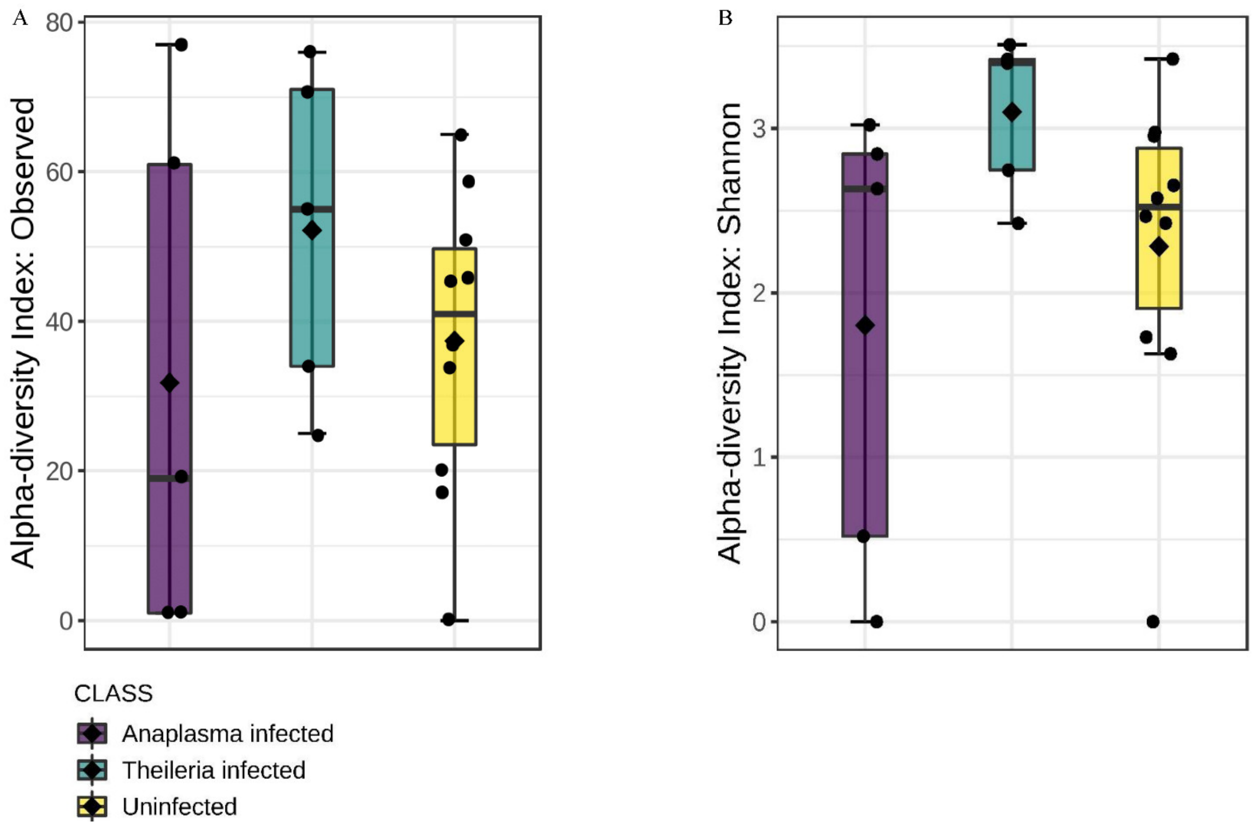

Figure S6. Alpha diversity analysis in *R. microplus* ticks**.** (A) Estimation of species richness using the observed operational taxonomic (OTUs) metrics (Kruskal-Wallis H-test, df = 1, p-value=0.434). (B) Estimation of species evenness using the Shannon diversity index (Kruskal-Wallis H-test, df = 1, p-value=0.165).

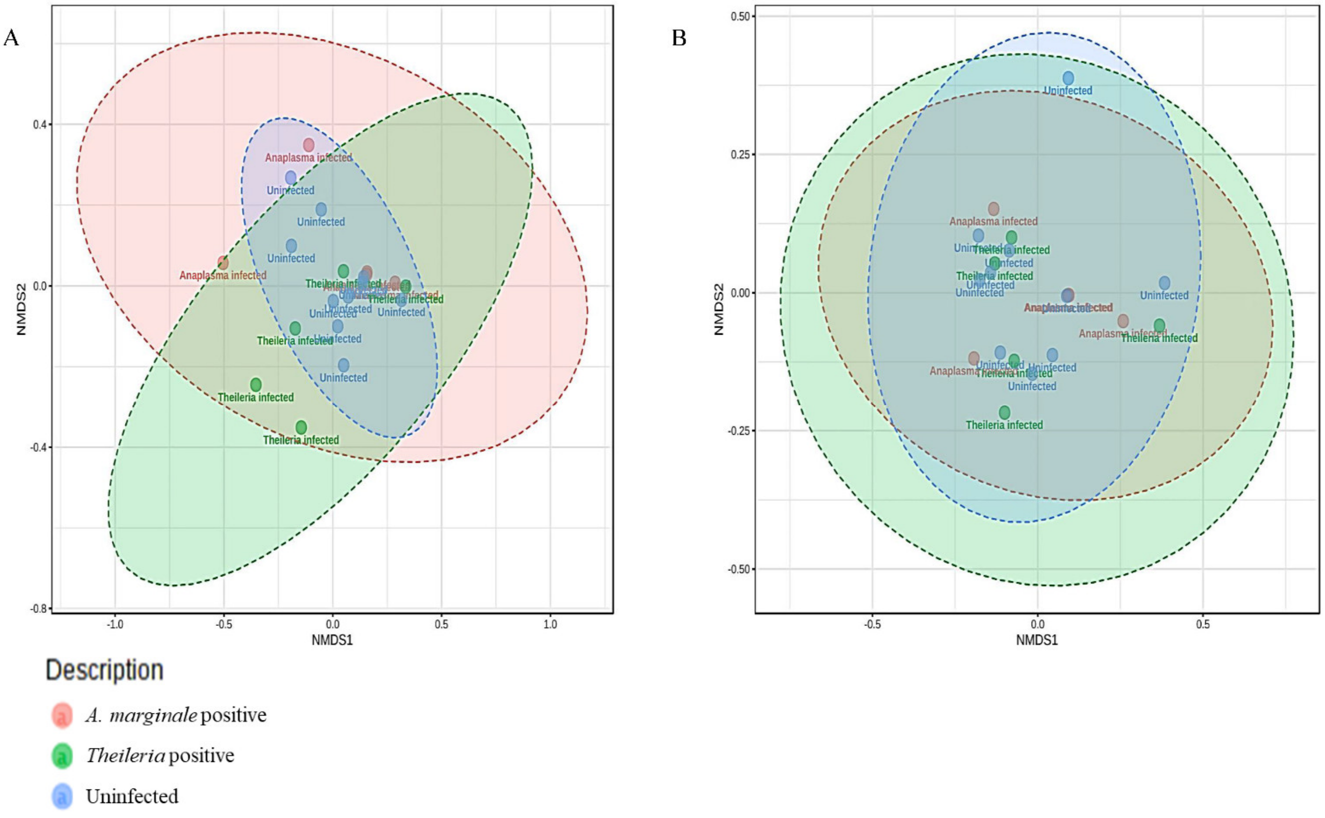

Figure S7: Visualization of differences in the microbial communities using Principal coordinate analysis (PCoA) of (A) Bray_Curtis and (B) Jaccard distance matrix in H. *anatolicum* ticks. No significant differences was observed in the clustering and distance analysis (PERMANOVA F-value: 1.0477; R: 0.10974; p-value = 0.404).

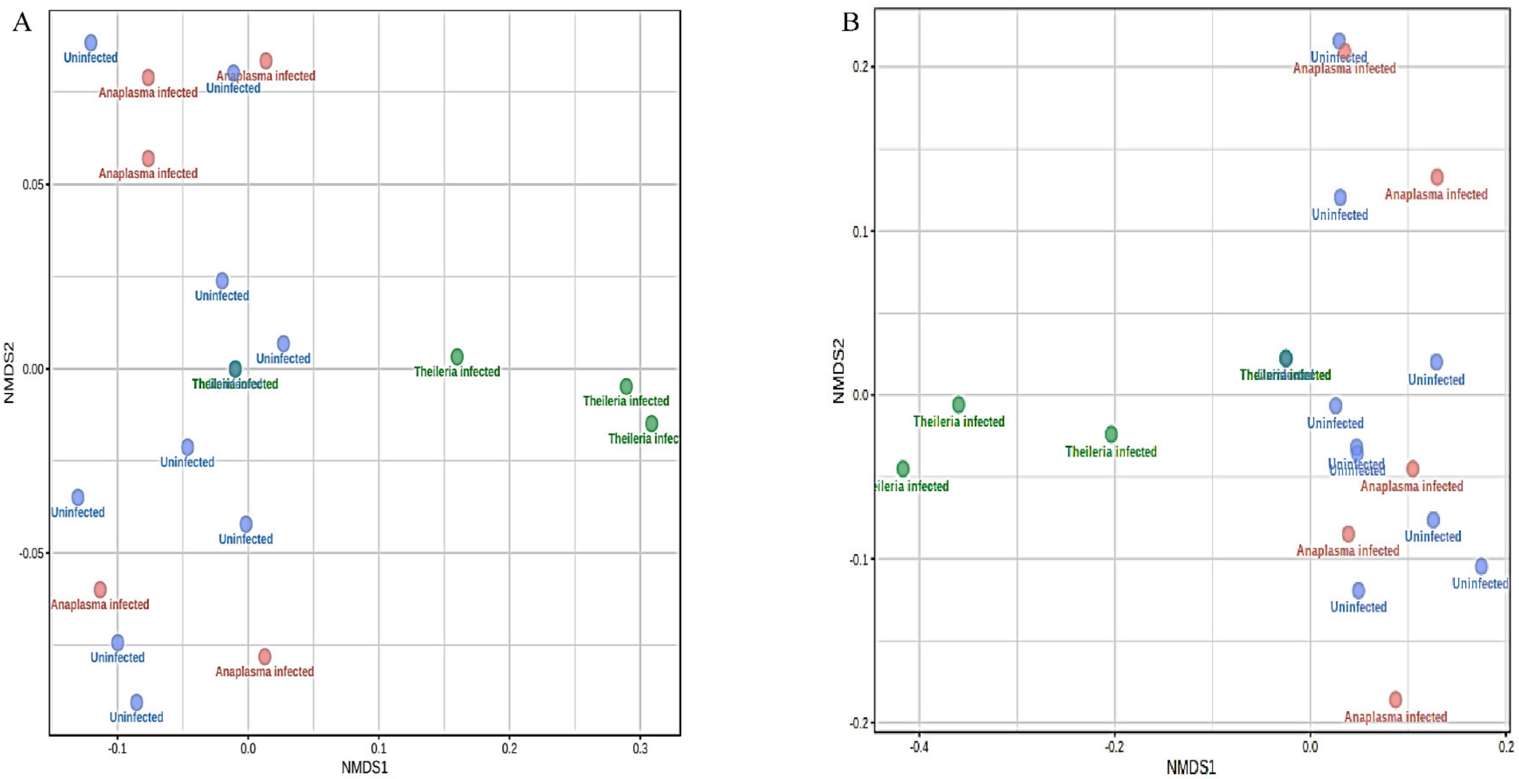

Figure S8: Non-metric multidimensional scaling (NMDS) plot of (A) Bray_Curtis (PERMANOVA, F-value: 2.7147; R-squared: 0.24207; p-value < 0.008, (NMDS) Stress = 0.11556), and (B) Jaccard (PERMANOVA, F-value: 6.0624; R-squared: 0.4163; p-value < 0.021, (NMDS) Stress = 0.033433) distance matrixes in R. microplus ticks.

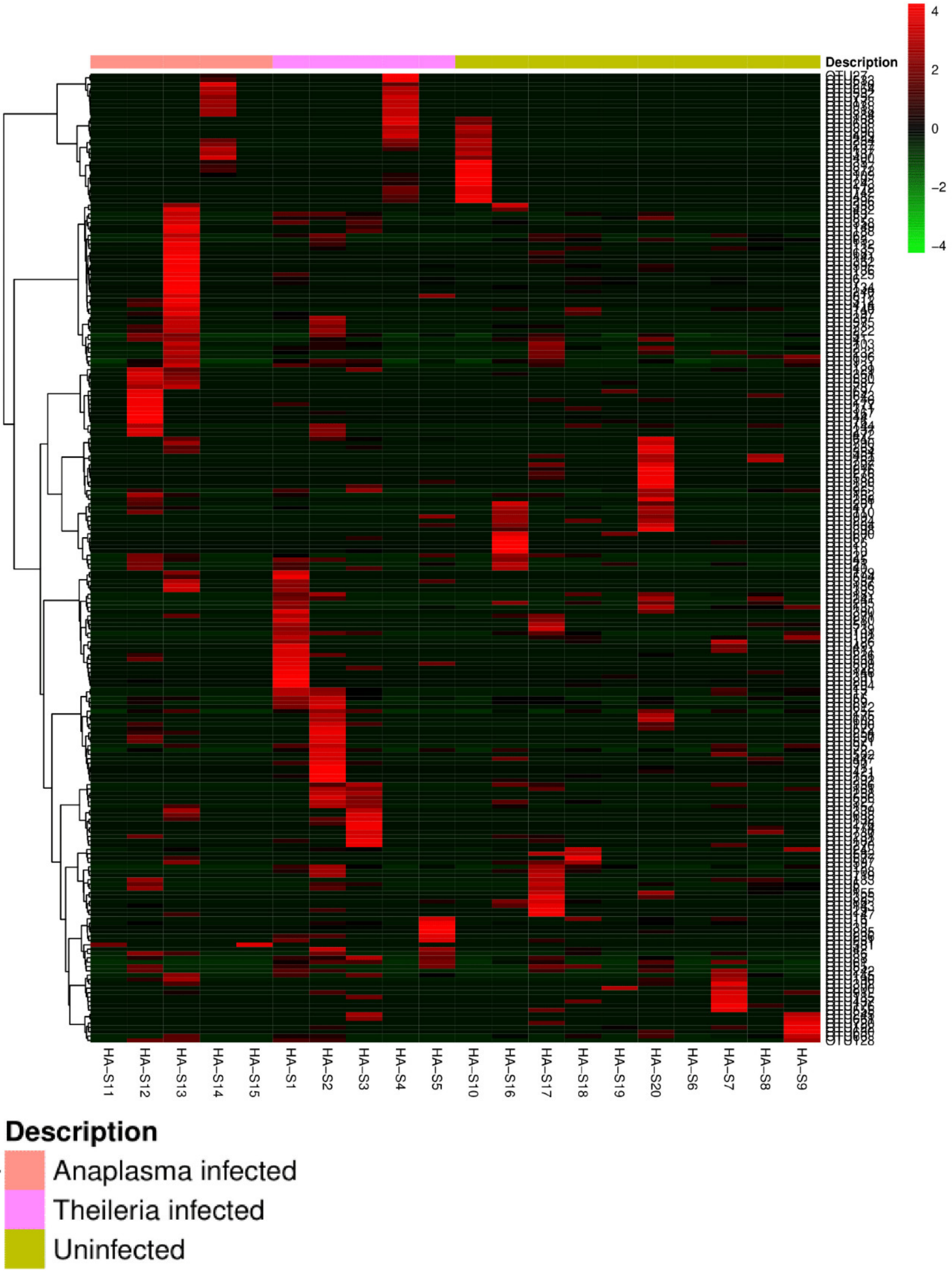

Figure S9: Heat map clustering of bacterial microbiota profile in *H. anatolicum* ticks
